# Lurasidone: a novel antipsychotic agent for the treatment of schizophrenia and bipolar depression

**DOI:** 10.1192/pb.bp.114.048793

**Published:** 2015-10

**Authors:** Antony Loebel, Leslie Citrome

**Affiliations:** 1 Sunovion Pharmaceuticals, Fort Lee, New Jersey, USA; 2 New York Medical College, Valhalla, New York, USA

## Abstract

Lurasidone is a novel antipsychotic agent approved for the treatment of schizophrenia in a number of countries including the UK, and is also approved in the USA and Canada for the treatment of major depressive episodes associated with bipolar I disorder as either a monotherapy or adjunctive therapy with lithium or valproate. In addition to full antagonist activity at dopamine D_2_ (*K*_i(D2)_ = 1 nM) and serotonin 5-HT_2_A (*K*_i(5-HT_2_A)_ = 0.5 nM) receptors, the pharmacodynamic profile of lurasidone is notable for its high affinity for serotonin 5-HT7 receptors (*K*_i(5-HT_7_)_ = 0.5 nM) and its partial agonist activity at 5-HT_1_A receptors (*K*_i(5-HT_1_A)_ = 6.4 nM). Long-term treatment of schizophrenia with lurasidone has been shown to reduce the risk of relapse. Lurasidone appears associated with minimal effects on body weight and low risk for clinically meaningful alterations in glucose, lipids or electrocardiogram parameters.

Lurasidone is a second-generation antipsychotic agent that initially received regulatory approval for the treatment of adults with schizophrenia in the USA in 2010.^1,2^ In March 2014, it received marketing authorisation for this indication by the European Medicines Agency and has also been approved for the treatment of schizophrenia in Switzerland, Canada and Australia. Additionally, lurasidone recently received US and Canadian regulatory approval for the treatment of adults with major depressive episodes associated with bipolar I disorder (bipolar depression), as either a monotherapy or adjunctive therapy with lithium or valproate. Detailed systematic reviews of the overall efficacy, tolerability, safety and place in therapy of lurasidone can be found elsewhere,^3,4^ including analyses of number needed to treat (NNT) and number needed to harm (NNH).^5,6^

The published literature has used the US convention of describing the dose of lurasidone as the combined weight of the active drug moiety (lurasidone) plus the hydrochloride (HCl) salt, with tablet strength expressed in multiples of 20 mg. In the European Medicines Agency *Summary of Product Characteristics*,^2^ tablet strength refers to the weight of active drug only, excluding the contribution from the HCl salt (Table 1 shows the dose equivalence), and this dose convention will be used in the current review.

**Table 1.** Lurasidone dose equivalents in the European Union (EU) and the USA

EU doses (mg, active moiety)	US doses (mg, HCl salt)
18.5	20

37	40

56^a^	60

74	80

111^a^	120

148^a^	160^b^

a. Tablet strength not available in the EU.

b. Tablet strength not available in the USA.

The purpose of this overview is to describe the pharmacodynamics and pharmacokinetics of lurasidone, and to summarise its efficacy and safety for the treatment of schizophrenia and bipolar depression based on results from both short-term and longer-term controlled clinical trials. Relevant information regarding switching and extension studies is reported, including functional and cognitive outcomes.

## Pharmacodynamics and pharmacokinetics

Similar to most other second-generation antipsychotic agents, lurasidone is a full antagonist at dopamine D_2_ and serotonin 5-HT_2_A receptors, with binding affinities *K*_i_ = 1 nM and *K*_i_ = 0.5 nM, respectively.^7^ In addition, lurasidone is distinguished by its high affinity for serotonin 5-HT_7_ receptors (*K*_i_ = 0.5 nM; comparable with dopamine D_2_ and 5-HT_2_A receptors) and by its partial agonist activity at 5-HT_1_A receptors (*K*_i_ = 6.4 nM).^7^ The serotonin 5-HT_7_ receptor is a target of interest that may be associated with the potential for both pro-cognitive and antidepressant effects,^8,9^ whereas the 5-HT_1_A receptor may have a role in the treatment of major depressive disorder^10^ and schizophrenia.^11^ Lurasidone lacks affinity for histamine H_1_ and muscarinic M_1_ receptors.

The pharmacokinetic profile of lurasidone is consistent with once-daily administration, with an elimination half-life of 20–40 h.^2^ Mean C_max_ and area under the curve (AUC) for lurasidone were approximately threefold and twofold greater, respectively, in a comparison of administration with food *v.* fasting.^12^ Based on these data, and results from clinical trials, it is recommended that lurasidone be taken once daily in the evening, with a meal or within 30 min after eating.^1,2^ Lurasidone absorption is independent of food fat content.^12^

### Metabolisation

Lurasidone is metabolised primarily via CYP3A4 and, consequently, its use is contraindicated in the presence of strong inducers and inhibitors of CYP3A4. In the presence of moderate inhibitors of CYP3A4, the recommended starting dose of lurasidone is 18.5 mg/day instead of the usual recommended 37 mg/day, and the highest recommended dose is 74 mg/day instead of the regular maximum recommended dose of 148 mg/day. Lurasidone does not affect the pharmacokinetics of other drugs including lithium, valproate or agents that are metabolised by the CYP3A4 pathway.^13^

Examination of population subgroups based on gender, age and ethnic background did not reveal any clear evidence of differential response to lurasidone,^1,2^ however, Asian subjects had one-and-a-half-fold increased exposure to lurasidone compared with White subjects.^2^ Clinical studies with lurasidone did not include sufficient numbers of patients aged 65 and older to establish whether dose adjustment is necessary on the basis of age alone. In patients with moderate or severe renal or hepatic impairment, the recommended starting dose is 18.5 mg/day; the maximum dose should not exceed 74 mg/day in moderate to severe renal impairment or moderate hepatic impairment and 37 mg/day in severe hepatic impairment.

## Efficacy in schizophrenia

### Acute treatment

Based on a robust registration programme that included five informative and similarly designed 6-week, fixed-dose, placebo-controlled studies,^14-18^ lurasidone is approved for the treatment of schizophrenia within a dose range of 37–148 mg/day. Reductions in the Brief Psychiatric Rating Scale^19^ or Positive and Negative Syndrome Scale (PANSS)^20^ total scores were consistently greater for lurasidone compared with placebo across the approved dose range. A starting dose of 37 mg/day has demonstrated significant efficacy, thus no initial dose titration is necessary. Subsequent dose increases can be made based on clinician judgement, typically in increments of 37 mg at approximately weekly intervals. Patients with suboptimal symptom control at lower doses of lurasidone may benefit from higher doses. The NNT of lurasidone (*v.* placebo) for a ≥30% reduction in PANSS total score was 4 (95% CI 3–5) for lurasidone 148 mg/day compared with 6 (95% CI 5–10) at 37 mg/day.^5^

The short-term effectiveness of lurasidone has also been evaluated in a study in which clinically stable but symptomatic out-patients with schizophrenia or schizoaffective disorder were switched from their current antipsychotic medication to lurasidone in a 6-week study that examined the efficacy and tolerability of three different dosing strategies (starting at 37 mg/day for 2 weeks, *v.* starting at 74 mg/day for 2 weeks, *v.* starting at 37 mg/day for 1 week followed by 74 mg/day the second week).^21^ The primary outcome was time to treatment failure, defined as any occurrence of insufficient clinical response, exacerbation of underlying disease or discontinuation due to an adverse event. No clinically relevant differences were observed among the three groups in efficacy or tolerability outcomes; treatment failure rates were low for all three switch groups (~8%).

### Long-term treatment

**Fig 1:**
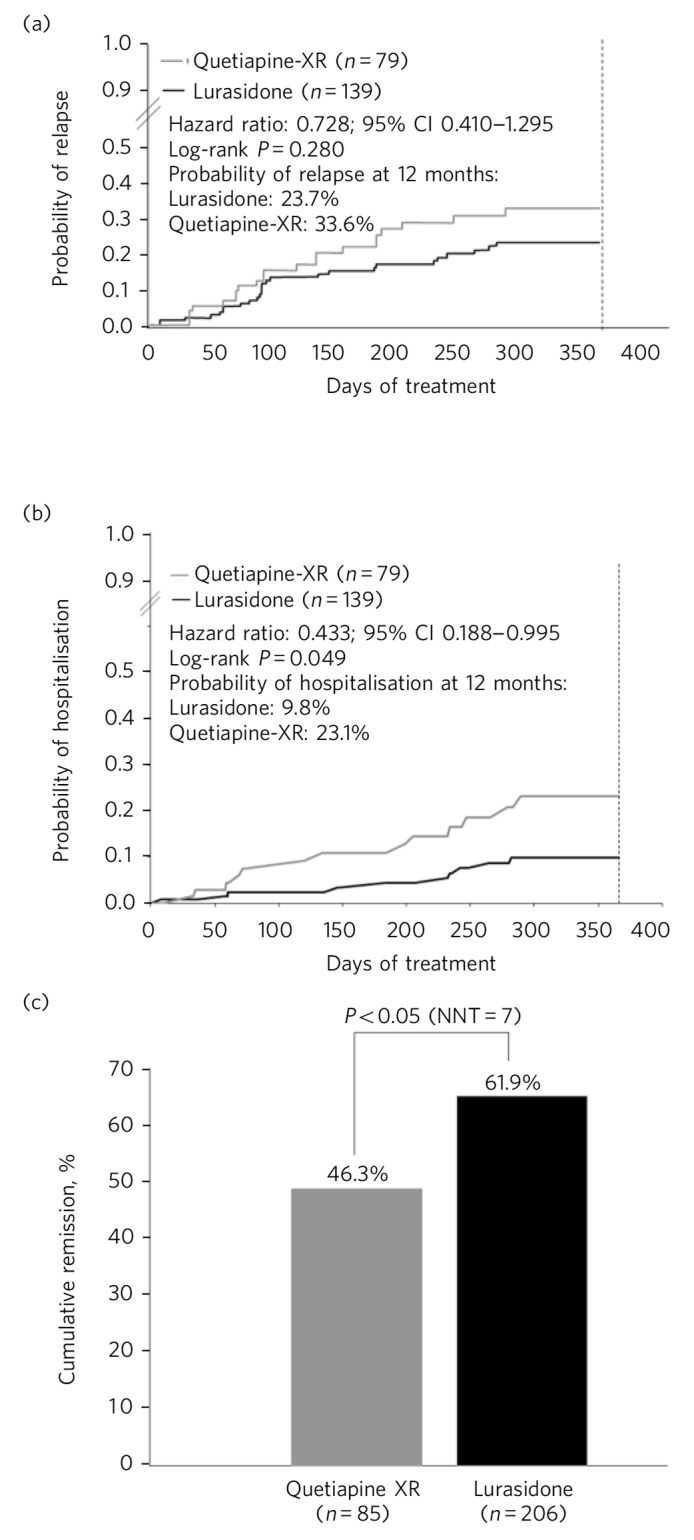
Kaplan–Meier estimates of the risk of relapse and rehospitalisation during 12 months of treatment with lurasidone *v*. quetiapine extended release (XR): a. probability of relapse; b. probability of rehospitalisation; c. cumulative remission.

Longer-term data for lurasidone in patients with schizophrenia are available based on randomised, double-blind, 12-month trials that include comparisons with risperidone in a safety study,^22^ and quetiapine extended-release (XR) in a double-blind extension to one of the short-term pivotal trials.^23^ In the risperidone comparator study, treatment with lurasidone was associated with comparable improvement in efficacy, with similar relapse rates at 12 months. In the quetiapine XR comparator study, lurasidone was non-inferior to quetiapine XR in risk for relapse. At 12 months, treatment with lurasidone (modal daily dose 111 mg) was associated with a significantly greater improvement in PANSS total score compared with quetiapine XR (modal daily dose 600 mg), numerically lower risk of relapse (23.7% *v.* 33.6%; *P* = 0.280; Fig. 1a), significantly lower risk of re-hospitalisation at 12 months (9.8% *v.* 23.1%; log-rank *P* = 0.049; Fig. 1b) and significantly higher rates of remission (61.9% *v.* 46.3%; *P* = 0.043; Fig. 1c). In the same study, a computerised cognitive battery (Cogstate; http://cogstate.com) was administered at the end of 6 weeks of acute double-blind treatment and after 6 months of double-blind extension treatment. At both time points, treatment with lurasidone was associated with significantly greater improvement in cognition compared with quetiapine XR, with a moderate effect size.^24^

The long-term effectiveness of lurasidone in the treatment of schizophrenia has also been evaluated in two open-label extension studies. In the first, a 6-month extension of the switch study summarised above,^25^ the mean PANSS total score continued to show improvement. Moreover, low rates were observed for both psychiatric emergency services utilisation (mean: 0.8% per month) and contact with the criminal justice system (mean: 1.8% per month).^25^ In a second open-label extension study in which patients received 6 months of treatment with lurasidone, antipsychotic efficacy was maintained, with further reduction observed in mean PANSS total scores, in patients who had initially received lurasidone, olanzapine or placebo during the acute treatment phase.^26^

Preliminary results of a double-blind, randomised withdrawal study examining the maintenance of efficacy of lurasidone treatment in patients with chronic schizophrenia have been presented.^27^ Patients experiencing an acute exacerbation of schizophrenia received flexible doses of lurasidone (37 or 74 mg/day) during a 12- to 24-week open-label stabilisation phase. Those who maintained clinical stability for ≥12 weeks entered a 28-week, double-blind withdrawal phase and were randomised to receive either lurasidone (at the same dose they were receiving at completion of the stabilisation phase) or placebo. Lurasidone significantly delayed time to relapse compared with placebo (log-rank test *P* = 0.039) and was associated with a 34% reduction in risk of relapse (Cox proportional hazard model ratio 0.66 (95% CI 0.45–0.98); *P* = 0.041).

### Tolerability and safety

Commonly observed adverse reactions in short-term trials in schizophrenia (incidence on lurasidone ≥5% and twofold greater than placebo) were somnolence (17% *v.* 7%; NNH = 10), akathisia (13% *v.* 3%; NNH = 10), nausea (10% *v.* 5%; NNH = 20) and extrapyramidal symptoms (excluding akathisia and restlessness) 14% *v.* 6% (NNH = 13).^1,2^ Akathisia and extrapyramidal symptoms appear dose related within the dose range of 18.5 to 111 mg/day.^1^ The frequency of akathisia in patients with schizophrenia was 5.6% for 18.5 mg, 10.7% for 37 mg, 12.3% for 74 mg and 22.0% for 111 mg. In a study where lurasidone was administered in the evening,^18^ akathisia was reported by 7.4% of patients receiving lurasidone 148 mg/day. It is possible that evening dose administration is associated with more favourable tolerability overall relative to morning dosing. Adverse event frequencies (including movement disorders) reported in the bipolar depression programme, where lurasidone was dosed at night in all studies, were generally lower than observed in patients with schizophrenia.^1^ The mean change in weight observed across 6-week trials in schizophrenia was +0.43 kg for lurasidone *v.* −0.02 kg for placebo.^1^ In contrast, mean change in weight was +4.15 kg for olanzapine and +2.09 kg for quetiapine XR in 6-week trials where these agents served as active controls.^1,17,18^ In 6-week trials, the proportion of patients with a clinically meaningful (≥7%) endpoint increase in body weight was 4.8% for lurasidone *v.* 3.3% for placebo (NNH = 67; not significant).^1^ In contrast, the proportion of patients with clinically significant weight gain during short-term treatment in one comparator study with olanzapine *v.* placebo was 34% *v.* 7% (NNH = 4);^17^ and the proportion of patients with clinically significant weight gain in a second comparator study with quetiapine XR *v.* placebo was 15% *v.* 3% (NNH = 8).^18^ The proportion of patients with clinically significant weight gain on lurasidone was similar to the rate for placebo in both comparator studies (NNH>55).^17,18^

Longer-term studies for lurasidone are consistent with short-term findings regarding changes in body weight; in a long-term study lurasidone was associated with a mean change in weight of +0.73 kg at month 12, compared with +1.23 kg on quetiapine XR.^2^

The short-term effect of lurasidone on metabolic variables appears minimal.^1^ In pooled short-term (6-week) clinical trials, the mean last observation carried forward (LOCF)-endpoint change in total fasting cholesterol was −0.15 mmol/L for lurasidone and −0.16 mmol/L for placebo;^28^ for fasting triglycerides it was −0.15 mmol/L for lurasidone and −0.17 mmol/L for placebo;^28^ and for fasting glucose it was +0.07 mmol/L for lurasidone and +0.03 mmol/L for placebo.^28^ Long-term data regarding metabolic outcomes extending out to 12 months are consistent with the short-term data.^1,28^ In long-term studies,^28^ mean LOCF-endpoint change on lurasidone was −0.08 mmol/L for total fasting cholesterol, −0.08 mmol/L for fasting triglycerides and +0.11 mmol/L for fasting glucose.

A moderate dose-dependent increase in prolactin was observed in patients treated with lurasidone, with more pronounced effects in female than in male patients; however, the increase was lower than what is observed with risperidone and haloperidol. In a randomised, 12-month, double-blind safety study comparing lurasidone with risperidone, mean change from baseline to endpoint in serum prolactin levels in men was +2.51 ng/ml for lurasidone and +9.45 ng/ml for risperidone, and in women it was +5.16 ng/ml for lurasidone and +33.90 ng/ml for risperidone.^22^

Serial electrocardiograms during short-term and long-term trials indicate that lurasidone, at doses as high as 558 mg/day, does not have a clinically meaningful impact on the QT interval.^1^

## Bipolar depression

There is an unmet need for efficacious and tolerable treatments for bipolar depression. Patients with bipolar disorder spend most of their symptomatic time in the depressed phase of their illness.^29^ While multiple agents are approved for the treatment of bipolar mania, there is a paucity of approved medications for the treatment of bipolar depression.^30^ The older interventions (quetiapine and olanzapine–fluoxetine combination) are as likely to provide therapeutic benefit as adverse effects.^30^ Cross-study comparisons in populations with bipolar depression suggest that treatment with lurasidone is associated with less sedation than quetiapine and less weight gain than the olanzapine–fluoxetine combination.^30^

Lurasidone, in the dose range of 18.5–111 mg/day, demonstrated superiority *v.* placebo in two 6-week, randomised, double-blind, placebo-controlled, flexibly-dosed acute studies in patients with major depressive episodes associated with bipolar I disorder, one using lurasidone monotherapy and the other using lurasidone adjunctive with lithium or valproate.^31,32^ As reported by Citrome and colleagues,^6^ monotherapy treatment with lurasidone (*v.* placebo) was associated with an NNT of 5 (95% CI 4–8) for treatment response, defined as ≥50% reduction from baseline on Montgomery-Åsberg Depression Rating Scale^33^ (MADRS) total score; adjunctive therapy with lurasidone was associated with an NNT of 7 (95%, CI 4–24). NNT for remission, defined as a final MADRS total score ≤12, was 7 (95% CI 4–14) for lurasidone monotherapy and 7 (95% CI 4–23) for adjunctive lurasidone. These results are comparable with NNT values reported for quetiapine (6 for response, 6 for remission) and olanzapine-fluoxetine combination (4 for response, 5 for remission).

Lurasidone was not associated with clinically meaningful mean weight or metabolic changes compared with placebo in these bipolar depression studies. This is in contrast to olanzapine-fluoxetine combination where the NNH (*v.* placebo) was 6 for clinically meaningful weight gain (≥7% from baseline).^6^ The three most frequently occurring adverse events with the largest difference in incidence for lurasidone *v.* placebo were nausea (NNH = 17 for monotherapy, NNH = 16 for adjunctive therapy), akathisia (NNH = 15 for monotherapy, NNH = 30 for adjunctive therapy) and somnolence (NNH = 25 for monotherapy, NNH = 19 for adjunctive therapy).^6^ The high NNH of lurasidone for somnolence compares favourably with the NNH of 3 (95% CI 2.2–2.7) reported for quetiapine in studies of bipolar depression, regardless of formulation or dose.^6^

Overall, the results of double-blind trials indicate that lurasidone has a highly favourable benefit/risk ratio for the treatment of bipolar I depression, with ‘single-digit’ NNT (indicating significant efficacy) and ‘double-digit’ or higher NNH (indicating high tolerability).

## Summary

Lurasidone represents a new addition to the pharmacological armamentarium available for the treatment of serious mental disorders. It has demonstrated efficacy in the treatment of schizophrenia, within the dose range of 37–148 mg/day, and bipolar depression, within the dose range of 18.5–111 mg/day. Across both indications, treatment with lurasidone appears associated with minimal effects on body weight and low risk for clinically meaningful alterations in glucose, lipids or ECG parameters. Lurasidone's combination of efficacy in schizophrenia and bipolar depression with minimal metabolic disturbance and little effect on movement disorders and prolactin represents a potentially important clinical advance.^34^
